# Automatic Segmentation and Classification for Antinuclear Antibody Images Based on Deep Learning

**DOI:** 10.1155/2023/1353965

**Published:** 2023-02-08

**Authors:** Qinghua Xie, Pengyu Chen, Zhaohuan Li, Renfeng Xie

**Affiliations:** ^1^The Affiliated Changsha Central Hospital, Hengyang Medical School, University of South China, Changsha, Hunan, China; ^2^International School, Beijing University of Posts and Telecommunication, Beijing, China

## Abstract

Antinuclear antibodies (ANAs) testing is the main serological diagnosis screening test for autoimmune diseases. ANAs testing is conducted principally by the indirect immunofluorescence (IIF) on human epithelial cell-substrate (HEp-2) protocol. However, due to its high variability and human subjectivity, there is an insistent need to develop an efficient method for automatic image segmentation and classification. This article develops an automatic segmentation and classification framework based on artificial intelligence (AI) on the ANA images. The Otsu thresholding method and watershed segmentation algorithm are adopted to segment IIF images of cells. Moreover, multiple texture features such as scale-invariant feature transform (SIFT), local binary pattern (LBP), cooccurrence among adjacent LBPs (CoALBP), and rotation invariant cooccurrence among adjacent LBPs (RIC-LBP) are utilized. Firstly, this article adopts traditional machine learning methods such as support vector machine (SVM), k-nearest neighbor algorithm (KNN), and random forest (RF) and then uses ensemble classifier (ECLF) combined with soft voting rules to merge these machine learning methods for classification. The deep learning method InceptionResNetV2 is also utilized to train on the classification of cell images. Eventually, the best accuracy of 0.9269 on the Changsha dataset and 0.9635 on the ICPR 2016 dataset for the traditional methods is obtained by a combination of SIFT and RIC-LBP with the ECLF classifier, and the best accuracy obtained by the InceptionResNetV2 is 0.9465 and 0.9836 separately, which outperforms other schemes.

## 1. Introduction

Antinuclear antibodies (ANAs) are autoantibodies that bind to human proteins of the cell nucleus. Identification of ANA patterns is an important tool for diagnosing autoimmune diseases such as systemic lupus erythematosus (SLE), scleroderma (SSc), polymyositis/dermatomyositis (PM/DM), and rheumatoid arthritis (RA), which are uncommon compared to other diseases but affect millions of people's health worldwide. Indirect immunofluorescence (IIF) using HEp-2 cells is one of the most common tests to detect ANAs. With the pattern of the HEp-2 cells shown under the microscope, doctors can learn therapeutically useful information and direct antigen-specificity tests in the future [1]. However, manual analysis of IIF images has significant drawbacks, including the subjectivity of results, long time consumption, inconsistency among laboratories, and low efficiency in processing a high number of cell images [2].

To make things better, computer-assisted pattern recognition techniques have been created in recent years to alleviate the strain of manual annotation and classification. EUROPattern [3] generates the detection outcome automatically with high precision, but the cost of using these softwares and systems are sometimes high, which increases the cost of the hospitals, especially for small and medium-sized hospitals[4]. With the help of traditional segmentation and classification methods and deep learning methods, a detection system with high classification accuracy rates and low implementation cost is expected.

Although previous studies have proposed several methods for the automatic segmentation of ANA cells and identification of ANA patterns, the overall framework of automatic segmentation and identification of ANA cell images with high accuracy and low complexity has not been developed.

Therefore, we design and implement an automatic scheme which can segment the given IIF images and classify the cell images gained from segmentation according to the ANA types. Firstly, the IIF images are segmented into 4 pieces separately to augment the dataset. The red and blue channels of IIF images will be removed to reduce the noise from the data. Secondly, the Otsu thresholding method [5] and watershed segmentation [6] algorithms are implemented to segment the ANA cells. Traditional machine learning classification methods (such as SVM) [7] k-nearest neighbours algorithm (KNN) [8], random forest (RF) [9], and the ensemble classifier (ECLF) [10] utilize various image features including scale-invariant feature transform (SIFT) [11], local binary pattern (LBP) [12], co-ccurrence among adjacent LBPs (CoALBP) [13], and rotation invariant cooccurrence among adjacent LBPs (RIC-LBP) [13] are compared to find the optimal combination of the learning method and features. Finally, the deep learning method InceptionResNetV2 is used for classification due to the highest accuracy shown in [14]. The transfer learning with the pretrained model is also used to improve the performance.

The designed scheme is tested for two datasets. One dataset comes from the practical medical images provided by the Changsha Central Hospital, which contains 126 IIF images after removing unusable pictures. This dataset contains two kinds of ANA patterns, which are Homogeneous and Speckled [15]. Because the number of classes is not enough to show the ability of the classification methods. Furthermore, the other dataset, ICPR 2016 open dataset is used, which contains six classes including Homogeneous, Speckled, Centromere, Golgi, Nucleolar, and NuMem [15]. This dataset contains 13596 cell images which are already segmented from the IIF images. The performance on the Changsha dataset is implemented to test the overall performance of the segmentation and classification. On the other side, performance on ICPR 2016 dataset is also done to show the performance of the classification methods and make comparisons between methods provided by prior articles. The final classification results from both datasets overperform the state-of-art level.

The contribution of this work could be summarized as follows:

1. We designed a segmentation and classification scheme for a given IIF image in order to classify automatically segmented cell images according to ANA types. It included IIF image segmentation, a traditional machine learning classification method to compare rotation invariant cooccurrence between adjacent LBPS (RIC-LBPs). Eventually, we used deep learning for classification. On this basis, a transfer learning method based on a pretrained model is proposed.
2. We tested the designed scheme on two datasets, one of which is the real medical images provided by Changsha Central Hospital and the other is the ICPR 2016 dataset. The final classification test results of the two datasets show that our classification method outperforms the methods provided in the existing articles.

The remainder of the article is organized as follows: in Section 2, the Otsu thresholding method, watershed segmentation algorithm, the concepts of SIFT, LBP, CoALBP, RIC-LBP, SVM, KNN, RF, and ECLF with the soft voting rule, and InceptionResNetV2 are explained and introduced. The training procedure is described in Section 3. In Section 4, the results, especially the accuracy of different methods, are compared and discussed.  Section 5 gives a conclusion to the whole paper.

## 2. Related Work and Background

### 2.1. Cell Segmentation Methods

Otsu thresholding method [5, 16] is still commonly used to choose the threshold for non-parametric and unsupervised picture segmentation, including the segmentation of IIF images. The discriminant criteria chooses an appropriate threshold to split the image into cells and background region.

The watershed transformation [6] is one of the most reliable region-based methods for automatic and unsupervised segmentation. For complex IIF image segmentation challenges, the images are treated as 3D topographic surfaces in the same way. The elevation at the relevant place is represented by the intensity of a pixel in the image. Finding the watershed lines in a topographic surface is the goal of watershed transformation [17].

Otsu thresholding method determines the threshold of image binarization segmentation, which divides the image into the background and foreground parts without the influence of image brightness and contrast. Watershed transformation provides a method of image edge segmentation by finding the dividing line between regions. Therefore, this paper combines the above two methods for cell segmentation.

### 2.2. ANA IIF Image Features

After investigation, typical texture features (SIFT, LBP, CoALBP, and RIC-LBP) utilized in ANA pattern classification are included in the research.

SIFT technique [11] firstly performs feature detection in scale space, defines the key point positions and scale, and then uses the primary direction of the neighbourhood gradient of the key points as the direction features of the points. The LBP operator represents a local region as a binary pattern formed by thresholding the difference between a centre pixel and its nearby pixels in a local region [12].

The computation of LBP at the vector **r**=(*x*, *y*) in the image *I* can be expressed by the following equation:

(1) $$\begin{array}{c} LBP\left(r\right)=\overset{N-1}{\underset{i=0}{\sum }}sign\left(I\left(r+{∆s}_{i}\right)-I\left(r\right)\right)2^{i}\text{,} \end{array}$$

where *N* denotes the number of pixels in the immediate vicinity and ∆**s**_*i*_ is a displacement vector from the reference pixel to surrounding pixels supplied by ∆**s**_*i*_=(*s* cos*θ*_*i*_, *s* sin*θ*_*i*_), where *θ*_*i*_=(360°/*N*)*i* is a scale parameter of LBP and *s* is a displacement vector from the reference pixel. The LBP histogram feature is a histogram of LBPs over an entire image. In most cases, *N* is set to 8.

Because the LBPs are crammed into a single histogram, spatial interactions between them are often ignored throughout the LBP histogram production process. The LBP histogram feature has been expanded to the CoALBP histogram feature, which incorporates information on cooccurrence among the LBPs, to analyse the spatial relationship among the LBPs [13].

The equation for a pair of two LBPs at different places is as follows:

(2) $$\begin{array}{c} P\left(r,{∆r}_{\phi }\right)=\left(LBP\left(r\right),LBP\left(r+{∆r}_{\phi }\right)\right)\text{,} \end{array}$$

where ∆**r**_*ϕ*_ is a displacement vector between two LBPs, and it is defined as (*r* cos *ϕ*, *r* sin *ϕ*), where *r* is the distance between two LBPs, and *ϕ*=0, 45°, 90°, 135°. Four different displacement vectors are used to create four different types of LBP pair configurations. By using this method, four histograms can be extracted from an image [12].

Because rotation invariance is a key feature of HEp-2 cell categorization, RIC-LBP [13] with both rotation invariance and good descriptive ability is better at handling this challenge. Rotation invariance is ensured in RIC-LBP by labelling each LBP pair with a rotation-invariant label. The following process is used to label the items. First, an LBP pair's shape is rewritten as follows:

(3) $$\begin{array}{c} P_{\phi }\left(r,{∆r}_{\phi }\right)=\left({LBP}_{\phi }\left(r\right),{LBP}_{\phi }\left(r+{∆r}_{\phi }\right)\right)\text{,}\\ {LBP}_{\phi }\left(r^{\prime }\right)=\overset{N-1}{\underset{i=0}{\sum }}sgn\left(Ir^{\prime }\left(+{∆s}_{i,\phi }\right)-I\left(r^{\prime }\right)\right)2^{i}\text{,}\\ r^{\prime }=r\;or\;r+{∆r}_{\phi }\text{,}\\ {∆s}_{i,\phi }=\left(s\;\cos\;\left(\theta_{i}+\phi \right),s\;\sin\;\left(\theta_{i}+\phi \right)\right)\text{,} \end{array}$$

where the rotation angle of a whole LBP pair is indicated by *ϕ*. When their binary patterns are rotation equivalent, the same label is assigned to *P*(**r**, ∆**r**_*ϕ*_)(*ϕ*=0, 45°, 90°, 135°, and 180°) to obtain rotation invariance.

As a result, the dimension of RIC-LBP is much smaller than that of CoALBP. Furthermore, RIC-LBP assumes that rotation angles are in 45° increments and that RIC-LBP is invariant in these angles. As a result, rotational robustness can be simply improved. Furthermore, simple methods such as thresholding and histogram calculation can be used to obtain the RIC-LBP histogram. As a result, RIC-LBP can be retrieved with minimal computing effort [13].

### 2.3. AI Methods for ANA Pattern Classification

After the features are subtracted, machine learning and deep learning methods are employed to determine the ANA pattern.

SVM is a sophisticated machine learning algorithm that has been successfully used for a variety of tasks, including classification, regression, and other tasks [7]. The classification is based on the identification of the maximum-margin hyperplane that divides the given training instances in this high-dimensional space, as well as the implicit mapping of data to a higher-dimensional space via a kernel function. Based on the class labels indicated by the k-closest neighbours of the vectors, the KNN decision process provides a straightforward nonparametric procedure for assigning a class label to the input pattern. Random forest is an ensemble learning method for classification that works by building a large number of decision trees in training. The method entails creating several trees in randomly chosen subspaces of the feature space. Trees in various subspaces generalize in complementary ways, and their combined categorization can be improved monotonically [9].

Based on the classifiers, the argmax of the sums of the predicted probabilities predicts the class label in a soft voting classifier, which is suggested for an ensemble of well-calibrated classifiers.

In addition, six different deep learning methods, including InceptionResNetV2, MobileNetV2, Xception, VGG19, ResNet50V2, and DenseNet121 are implemented and compared in the classification of IIF images in [14]. With pretrained models, InceptionResnetV2 achieves the highest *F*1 score of 0.86, so InceptionResnetV2 is chosen as the deep learning method in this paper.

The inception deep convolutional architecture, often known as GoogLeNet or Inception-v1, was first introduced in [18]. Later, the inception architecture was enhanced in several ways, the first of which were Ioffe and Szegedy introduction of batch normalization [19] (Inception-v2). Additional factorization ideas were later added to the architecture in the third iteration [20] which is referred to as Inception-v3 in [21].

**Figure 1:**
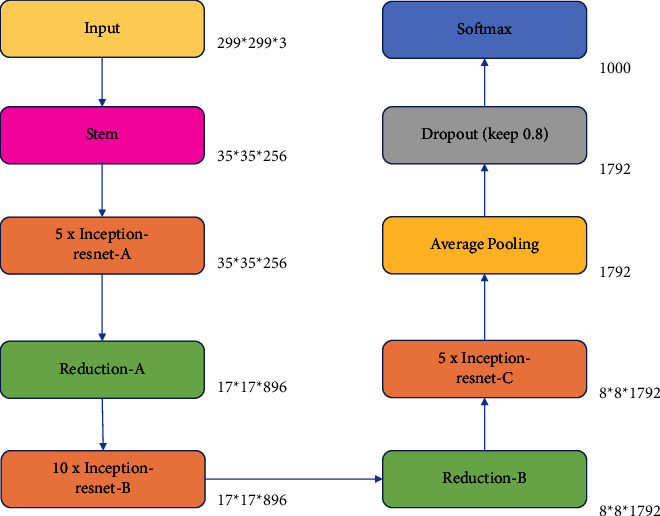
Structure of InceptionResNetV2.

The version of Inception-ResNet-v2 is a more expensive hybrid version with much-enhanced recognition performance [22]. The structure of InceptionResNetV2 is shown in Figure 1. The inception architecture has been proved to deliver excellent results at a low computational cost. In the 2015 ILSVRC challenge, the addition of residual connections in conjunction with a more traditional architecture resulted in a great performance, which was comparable to the Inception-v3 network. This begs the question of whether combining the Inception design with residual connections is beneficial. In [21], it is demonstrated that training with residual connections greatly speeds up the training of inception networks. There is a further evidence that residual inception networks outperform similarly priced inception networks with no residual connections by a small amount.

### 2.4. Related Research Progress on ANA Detection

In [23], Li et al. segmented the IIF images of their dataset with block segmentation, extracted LBP, SIFT, LDA, and GLCM from the blocks, and then used SVM, KNN, and BPNN to classify these features. The final output were determined by a fusion of the results gotten with the blocks from the same image based on some different fusion rules. Multiple combinations of features, classifiers, and fusion rules were adopted to find the best one, which was the LBP + KNN + majority rule, which achieved an accuracy of 94.62%.

In [24], the authors implemented RIC-LBP combined with a motif pattern cooccurrence labels (MCLs) as the feature and used random forest as the classification method, which got an accuracy of 0.9426 on the ICPR 2016 dataset. In [25], the writers used features extracted with VGG-19, RIC-LBP descriptor, and joint motif labels (JMLs) descriptor combined with random forest classifier, which reached an accuracy of 0.9211 on the HEp-2 specimen benchmark dataset.

Deep learning methods were also widely adopted in this area. Jia et al. [26] presented a network based on convolutional neural network (CNN) and got an accuracy of 0.9826 on the ICPR 2016 HEp-2 training set with data augmentation. Lei et al. [27] adopted ResNet50 to extract features and classify the images, which got an accuracy of 0.9842. Nigam et al. [28] designed and built multitask generative adversarial networks (GANs) to get accurate segmentation masks of the cells for better classification performance and used ResNet-34 and MobileNetv3 for segmentation and classification, which achieved an accuracy of 0.9882 for the classification on the ICPR 2016 dataset.

## 3. Design and Implementation

**Figure 2:**
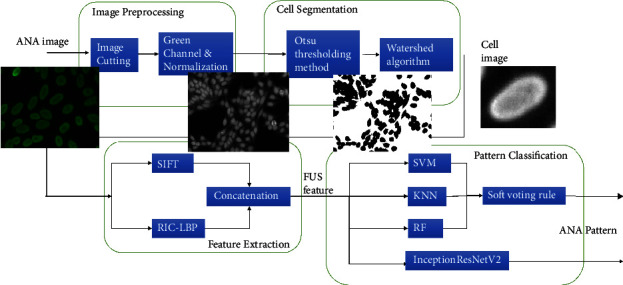
Segmentation and classification framework.

As described in Sections 1 and 2, both traditional machine learning methods and deep learning methods are used in this article, which are the key of the article. In machine learning and deep learning methods, feature selection and fusion are the keys to improve classification accuracy. So, we design a unified segmentation and classification framework in order to improve the precision as Figure 2.

**Figure 3:**
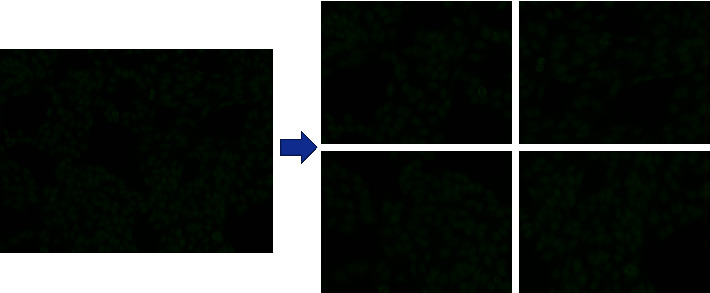
The IIF image is divided into four parts.

Firstly, the raw IIF images will be cut into 4 images as shown in Figure 3. Then, these images will be separated into train set and test set in a ratio of 8 : 2. Moreover, after that, the images will be preprocessed, including removing red and blue channels and normalization.

Then, the images will be segmented into cell images using the Otsu thresholding method and watershed segmentation algorithm in sequence.

For traditional machine learning methods, SIFT and three versions of LBP (LBP, CoALBP, and RIC-LBP) feature extraction will be adopted. Moreover, all four features will be used to train the classifier and show the results on the test set. The LBP feature with the best output will be concatenated with SIFT feature to get the final feature to be used. As told in Section 2, SVM, KNN, RF, and ECLF with the soft voting rule will be used on the features to train and generate the model to show the efficiency of classification.

For the deep learning method, InceptionResNetV2 is used. Moreover, to boost the final result, the homogeneous and speckled cell images in the ICPR 2016 dataset will be used to get the pretrained model. Then, based on the pretrained model, the InceptionResNetV2 model will be trained on the Changsha dataset. Finally, the performance will be tested on the test set.

### 3.1. Cutting and Preprocessing

The IIF image is divided into four images as shown in Figure 3. The algorithm uses OpenCV and os packets, which will traverse the given directory and store the cut images in another given directory.

**Figure 4:**
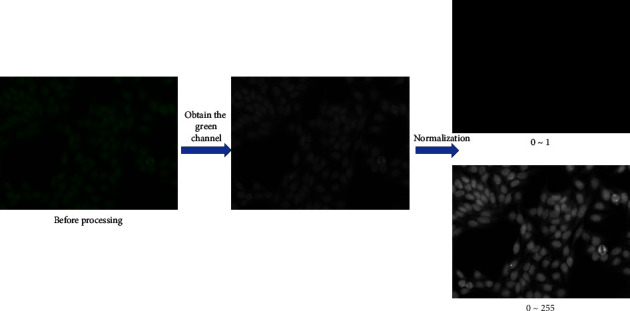
Image preprocessing process.

Then, the cut images will be preprocessed as shown in Figure 4. The images will be read with an OpenCV packet, and then the green channel will be obtained and stored in the given directory. Then, the green channel image will be normalized using the normalization function in OpenCV, and we can get the normalized image on the scale of 0∼1. By multiplying with 255, we get the normalized image on the scale of $0\sim255$. Moreover, they will be stored in the corresponding directory.

### 3.2. Cell Segmentation

**Figure 5:**
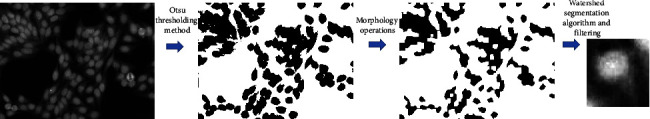
Process of using Otsu thresholding method and watershed segmentation algorithm.

Otsu thresholding method and watershed segmentation algorithm will be used in sequence as shown in Figure 5. First, the image will be blurred using a Gaussian filter to remove noises. Moreover, with the threshold function the in OpenCV packet, we can adopt the Otsu thresholding method, from which we can get the binary image of the input image. Then morphologyEx function in OpenCV is adopted to erode first and then dilate the binary image, which can remove the noises. Moreover, then dilate and distanceTransform function in OpenCV are adopted to get the sure foreground area and the sure foreground area. By removing the sure area from the image, we get the unknown region. Then, we can adopt the watershed method in OpenCV to separate the unknown region into background or foreground. After separation, the boundary will be marked, and then the regions with a diameter between 30 to 120 pixels, the centre locating 20 pixels or more from the margin of the image, and the brightness of the centre are over 50 will be stored as cell images which will be cut as rectangles.

### 3.3. Feature Extraction

Before extracting SIFT feature, the cell image will be resized to 128 *∗* 128, and the output will be an array with 128 values. As said in Section 2, to extract the LBP feature it is needed to determine the radius of the circle and the number of neighbours to be considered on the circle. Here, radius is set to 1 and 8 neighbours for one single point are found.

In CoALBP, it also implements 1 as the radius and 8 as the number of neighbours, and the radius for the cooccurrence of the patterns is set to 2.

As for RIC-LBP, LBP's radius is set to *s* = 1, 2, and 4 pixels, while LBP pair intervals are set to *r* = 2, 4, and 8 pixels. The features recovered by each parameter are then concatenated into a final recommended feature vector with a dimension of 408=(136*∗*3).

### 3.4. Traditional Machine Learning Methods

SVM, KNN, RF, and ECLF can be easily implemented with the help of the sklearn packet. The parameters are adjusted to each feature to get the best result. In SVM, the radial basis function is used as the kernel function for efficiency and accuracy. In KNN, the inverse of the distance is the weight of the points. Moreover, the number of trees in RF is 1000 for all the features.

### 3.5. Deep Learning Method

InceptionResNetV2 is implemented with PyTorch in this article. To have a better performance, it is pretrained on the ICPR 2016 dataset for 5 loops at the current learning rate of 0.0001, and the batch size is set to 16. After pretraining, the pretrained model will be adopted on the Changsha dataset, and it will be trained 30 times at the original learning rate of 0.00001, 0.00002, and 0.00003, and the batch size is set to 16.

## 4. Results and Discussion

In this section, the results and discussion will be presented in the sequence of three sections.

### 4.1. Training on the Changsha Dataset

After cutting, preprocessing, and cell segmentation, there are 3049 homogeneous cell images and 2930 speckled cell images in the training set, and 1033 homogeneous cell images and 733 speckled cell images in the test set. The ratio between the train set and test set is 2.95 and 3.99 separately.

#### 4.1.1. Feature Extraction and Traditional Machine Learning Method Training

After training and testing, the results of traditional machine learning methods trained with different features are shown in Table 1. It can be found that the ensemble classifier (ECLF) with the soft voting rule can increase the accuracy of the results, so it is adopted as the final machine learning method. According to the LBP features, RIC − LBP has the highest accuracy, so it is adopted. And to further increase the accuracy, SIFT feature and RIC − LBP feature are concatenated, which is named FUS with a length of 536 for each image. And FUS + ECLF obtained the highest accuracy on the test set, which is 0.9269. To show further show the effectiveness, the value of precision, recall, and *F*1 score are shown in Table 2.

#### 4.1.2. Deep Learning Method Training

The precision, recall, and *F*1 score are shown in Table 3.

### 4.2. Training on the ICPR 2016 Dataset

To test the effectiveness, both traditional machine learning methods and deep learning method are adopted for the training on the ICPR 2016 dataset.

The results of traditional methods are shown in Table 4. The same result can be obtained by the use of FUS and ECLF, which gets the best accuracy. The other evaluation values are listed in Table 5.

In comparison, in [24] the writers adopt RIC − LBP + Motif labels as the feature and use RF as the classifier. Finally, they obtained an accuracy of 0.9426 on the ICPR 2016 dataset, which is lower than that in this paper.

With InceptionResNetV2, the accuracy of classification achieves 0.9836, which is state-of-art. Other values are shown in Table 6. Moreover, the comparative studies of this dataset with other machine learning methods are shown in Table 7.

## 5. Conclusion and Further Work

In this article, traditional machine learning classification methods and deep learning classification methods are designed and constructed by implementing preprocessing and cell segmentation. Training on the ICPR 2016 dataset shows the effectiveness of the classification module.

Specifically, the Otsu threshold method and watershed segmentation algorithm are used to successfully segment cells from IIF images. On the Changsha dataset, the accuracy of the FUS (SIFT + RIC − LBP) feature classified by ECLF (SVM + KNN + RF) reaches 0.9269, and the accuracy of InceptionResNetV2 after the pretraining model reaches 0.9465. It is recommended to use these two models in practice for classification prediction according to the actual computational power. Training on the ICPR 2016 dataset shows that the entire classification module is state-of-the-art. The accuracy of FUS features classified by the ECLF classifier is 0.9635, and that of InceptionResNetV2 is 0.9836.

In future work, the proposed algorithm can be applied to the field of oncology, clone analysis of cell cultures, or cell quantification in fluorescence images, thus effectively supporting the work of researchers. Firstly, in biological analysis, automatic cell/colony segmentation and counting is essential due to the large image sets. In experiments, a user-friendly, adaptive, and robust image processing/analysis method is needed due to the problems of image acquisition condition drift, background noise, and high variation of colony characteristics. For example, this paper can combine the AutoCellSeg algorithm [32] to allow users to correct the results through the graphical interface and increase the accuracy of the results. Secondly, in the measurement of survival fraction (SF) in clonogenicity analysis, this study can assist or replace manual counting of cell colony forming units to prevent manual counting errors and colony merging resulting in manual identification. Compared with the percentage of area covered by colony (ACC) used in SF quantification [33], the method proposed in this paper can improve the accuracy of SF quantification. Meanwhile, quantum inspired machine learning (QiML) [34] is used to greatly reduce the computational complexity and improve the accuracy, which is more applicable to large-scale data than the algorithm proposed in this paper. In future work, we can further improve our work by referring to the quantum inspired classifier proposed in this article.

## Figures and Tables

**Table 1:** Accuracy obtained by different features and traditional machine learning methods.

Features	Methods			
	SVM	KNN	RF	ECLF
SIFT	0.8525	0.8072	0.8417	0.8532
LBP	0.8221	0.8275	0.8640	0.8728
CoALBP	0.8539	0.8444	0.8566	0.8809
RIC-LBP	0.9039	0.8424	0.8613	0.9053
FUS (SIFT + RIC − LBP)	0.9107	0.8451	0.8978	0.9269

**Table 2:** Precision, recall, and *F*1 score obtained by FUS + ECLF.

ANA pattern	Precision	Recall	*F*1 score
Homogeneous	0.9139	0.9650	0.9388
Speckled	0.9476	0.8742	0.9094

**Table 3:** Precision, recall, and *F*1 score obtained by InceptionResNetV2.

ANA pattern	Precision	Recall	*F*1 score
Homogeneous	0.9472	0.9615	0.9543
Speckled	0.9456	0.9258	0.9356

**Table 4:** Accuracy obtained by different features and traditional machine learning methods.

Features	Methods			
	SVM	KNN	RF	ECLF
SIFT	0.8399	0.8463	0.8760	0.8888
RIC-LBP	0.8241	0.8578	0.8767	0.8953
FUS	0.9563	0.9134	0.9150	0.9635

**Table 5:** Precision, recall, and *F*1 score obtained by FUS + ECLF.

ANA pattern	Precision	Recall	*F*1 score
Centromere	0.9797	0.9797	0.9797
Golgi	0.9872	0.9094	0.9467
Homogeneous	0.9547	0.9708	0.9627
Nucleolar	0.9897	0.9696	0.9795
NuMem	0.9441	0.9531	0.9486
Speckled	0.9456	0.9596	0.9540

**Table 6:** Precision, recall, and *F*1 score obtained by InceptionResNetV2.

ANA pattern	Precision	Recall	*F*1 score
Centromere	0.9918	0.9858	0.9888
Golgi	0.9839	0.9646	0.9742
Homogeneous	0.9865	0.9843	0.9854
Nucleolar	0.9874	0.9937	0.9905
NuMem	0.9758	0.9862	0.9810
Speckled	0.9773	0.9754	0.9763

**Table 7:** Comparative study for the ICPR 2016 dataset.

ANA pattern	Description	Accuracy
Nigam et al. [28]	Texture features + SVM	0.7163
Wiliem et al. [29]	DCT features + SIFT + SVM	0.7491
Nosaka and Fukui [30]	LPB + SVM	0.7944
Xie et al. [31]	5 layers CNN	0.9676
This work	InceptionResNetV2	0.9836

## Data Availability

The public dataset is cited as [26] within the article. The private dataset is restricted.
